# Supplementary material for: The tight junction protein TJP1 regulates the feeding-modulated hepatic circadian clock
Source: Nat Commun. 2020 Jan 30;11:589. doi: 10.1038/s41467-020-14470-2 (PMC6992704; doi:10.1038/s41467-020-14470-2)
Supplement: Supplementary file 1 — Supplementary Information [file 41467_2020_14470_MOESM1_ESM.pdf]

Supplementary Information

The Tight Junction Protein TJP1 Regulates the Feeding-modulated Hepatic Circadian Clock

Liu et al.

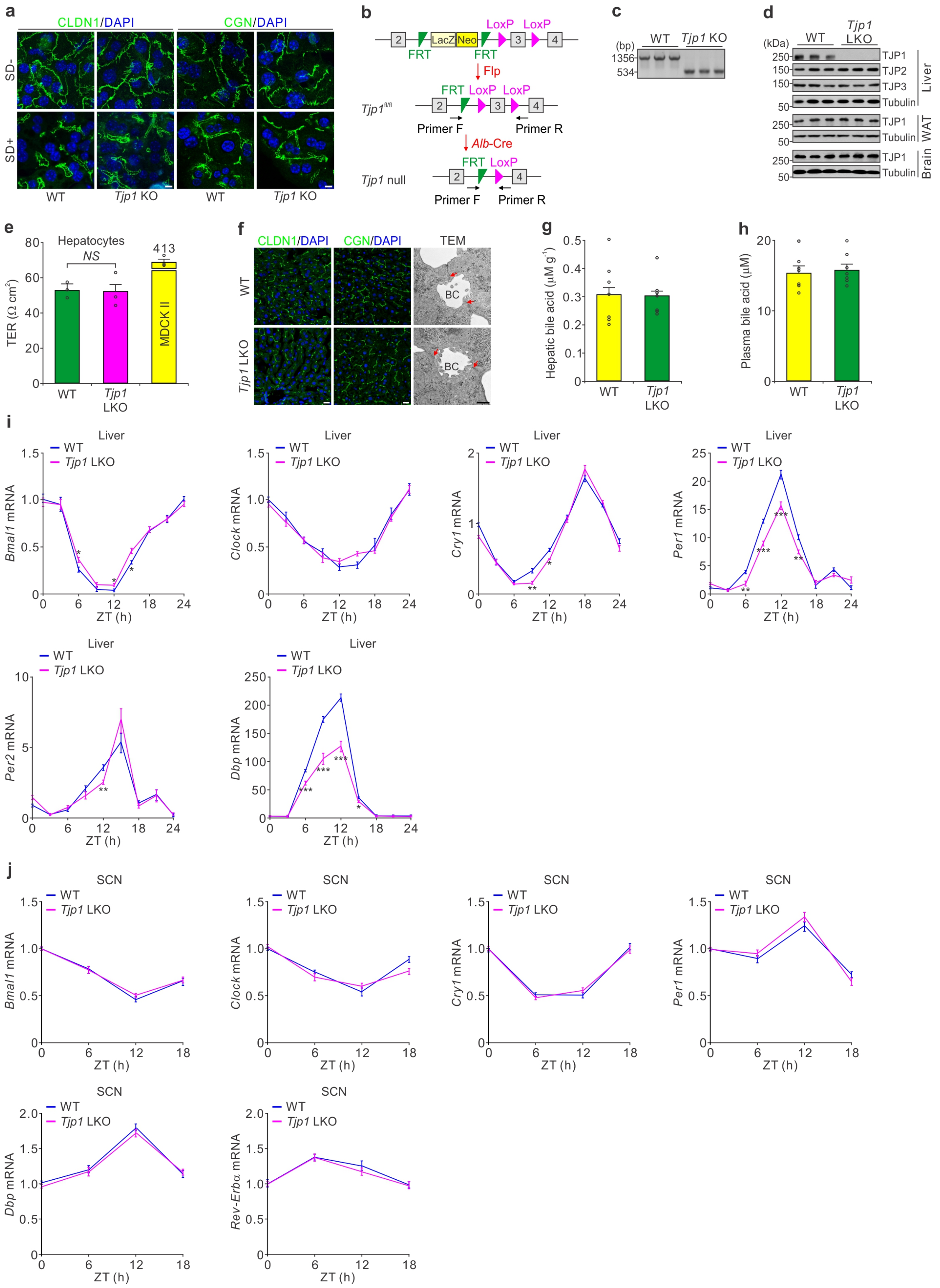

Supplementary Figure 1: Effect of *Tjp1* LKO on tight junctions and gene expression of core circadian components.

a CLDN1, CGN and DAPI staining in WT and *Tjp1* KO primary hepatocytes cultured in the presence (SD+) or absence (SD-) of a collagen sandwich configuration. Scale bars, 10 μm. **b** Generation of *Tjp1* LKO mice. A deletion of exon 3 was introduced into the *Tjp1* gene. The positions for genotyping primers

are shown. **c** Genotyping analysis showing *Tjp1* fragments generated from WT and *Tjp1* LKO mice. **d** Immunoblots showing the loss of TJP1 in liver extracts. WAT, white adipose tissue. **e-h** Tight junction formation evaluated by measurement of transepithelial electrical resistance (TER, **e**), CLDN1 or CGN staining and transmission electron microscopy (TEM, **f**), contents of bile acids in liver tissues (**g**) and levels of plasma bile acids (**h**) from WT and *Tjp1* LKO mice. TER for MDCK II cells is used as a control. Scale bar for CLDN1 staining, 20 μm; scale bar for TEM, 500 nm. BC, bile canaliculus. Red arrows in (**f**) indicate tight junctions. n = 7 mice. **i** qPCR results showing gene expression of core circadian oscillators in liver extracts from WT and *Tjp1* LKO mice. n = 3 ~ 7 mice. **j** qPCR results showing gene expression in suprachiasmatic nucleus (SCN) from WT and *Tjp1* LKO mice. n = 5 mice. Data are shown as mean ± s.e.m. Comparison of different groups was carried out using two-way ANOVA. $^*P < 0.05$, $^{**}P < 0.01$. *NS*, no statistical difference. Source data are provided as a Source Data file.

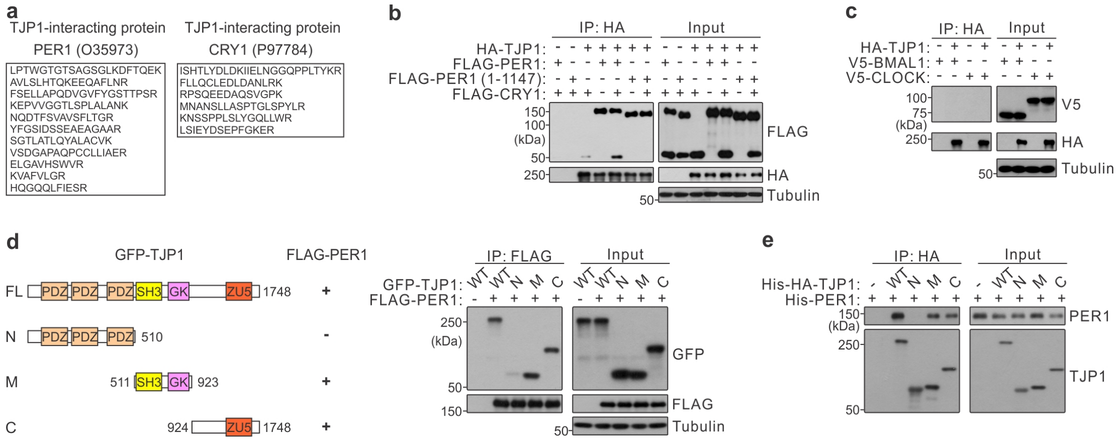

Supplementary Figure 2: Characterization of the interaction between TJP1 and PER1.

a The representative peptides identified from PER1 and CRY1 by MS analysis of immunoprecipitates obtained with anti-TJP1 antibody from SD+ cultured primary hepatocytes. **b** Immunoblots of co-IP assays showing amounts of FLAG-tagged PER1, PER1 mutant (1-1147 aa) or CRY1 recovered from immunoprecipitates of HA-TJP1 in HEK293T cells. **c** Immunoblots of co-IP assays showing amounts of V5-tagged BMAL1 or CLOCK recovered from immunoprecipitates of HA-TJP1 in HEK293T cells. **d** Deletion analysis of regions in TJP1 required for the TJP1-PER1 interaction in HEK293T cells. Interaction-competent TJP1 polypeptides are indicated by (+) in each schematic. **e** *In vitro* pull-down assay showing the binding ability of His-HA-tagged WT or mutants of TJP1 and His-PER1. Source data are provided as a Source Data file.

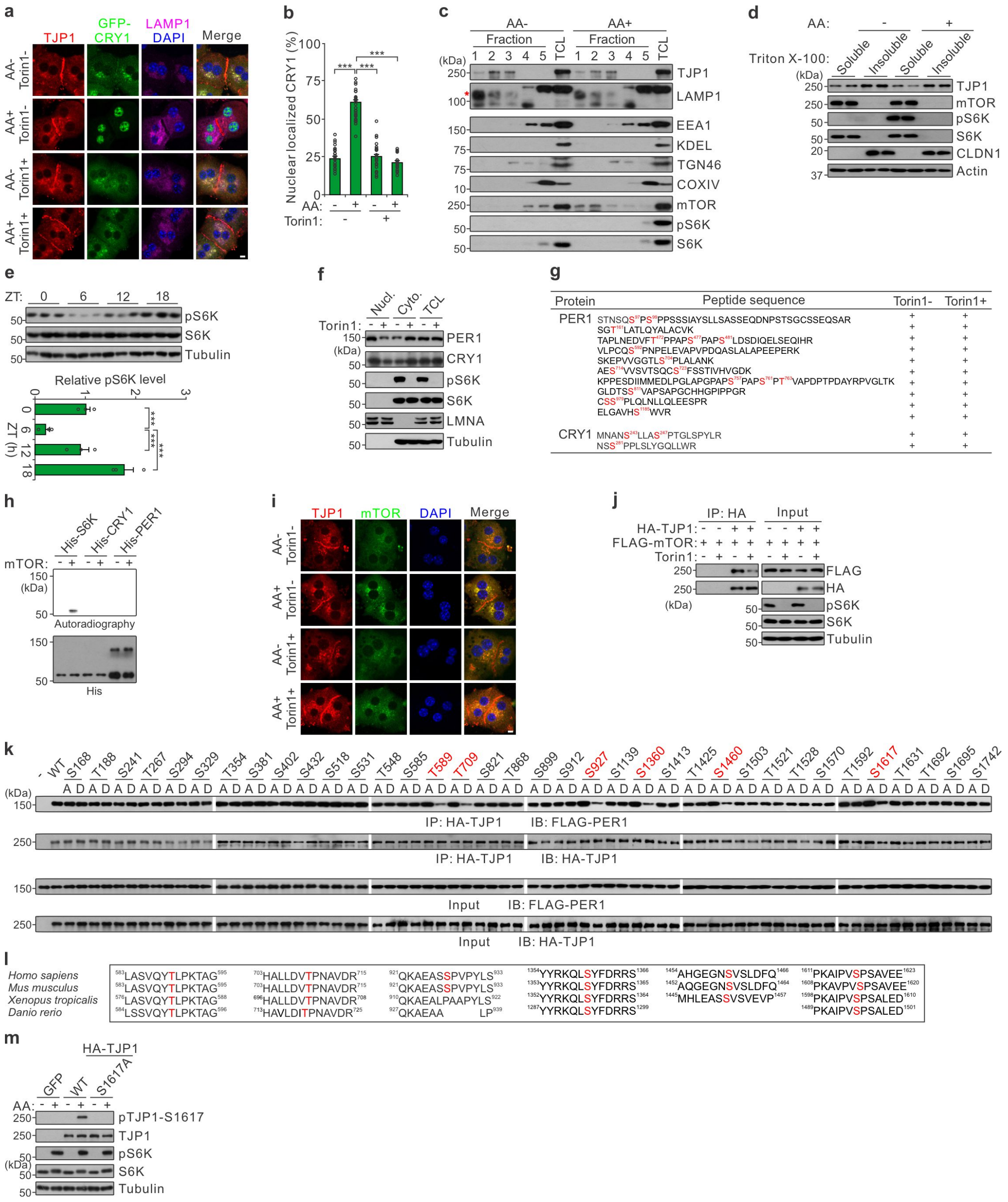

Supplementary Figure 3: Identification of mTOR phosphorylation sites on TJP1.

a-b Cellular localization of GFP-CRY1 and endogenous TJP1 (**a**) and statistical analysis of the results (**b**) in primary hepatocytes cultured in a collagen sandwich configuration in response to treatment with amino acids and/or Torin1. Mouse primary hepatocytes incubated with amino acid-free RPMI1640 for 6 hrs were exposed to 250 nM Torin1 or control vehicle for another 30 min, then treated with amino acids for 30 min. Scale bar, 10 μm. **c** Immunoblots showing the distribution of TJP1 in lysosome fractions. The red asterisk indicates LAMP1. Mouse primary hepatocytes incubated with amino acid-free RPMI1640 for 6 hrs were exposed to amino acids for 30 min. TCL, total cell lysate. **d** Immunoblots

showing the relative distribution of TJP1 in junction fraction (Triton X-100 insoluble) and cytosolic fraction (Triton X-100 soluble). Mouse primary hepatocytes incubated with amino acid-free RPMI1640 for 6 hrs were exposed to amino acids for 30 min. **e** mTOR activity evaluated by the level of pS6K in liver extracts of mice (top panel) and the relative level of pS6K (bottom panel). n = 6 mice. **f** Immunoblots showing the effect of Torin1 treatment on cellular localization of PER1 and CRY1 in SD+ cultured primary hepatocytes. **g** Phospho-peptides of FLAG-tagged PER1 and CRY1 identified by MS analysis of immunoprecipitates obtained with anti-FLAG from SD+ cultured primary hepatocytes treated with 250 nM torin1 for 1 hr (Torin1+) or not (Torin1-). **h** *In vitro* kinase assay showing the phosphorylation status of His-tagged S6K, CRY1 and PER1 by truncated mTOR. His-tagged S6K, CRY1 and PER1 were purified from *E coli*. **i** Cellular localization of endogenous TJP1 and mTOR in primary hepatocytes cultured in a collagen sandwich configuration in response to treatment with amino acids and/or Torin1. Mouse primary hepatocytes incubated with amino acid-free RPMI1640 for 6 hrs were exposed to 250 nM Torin1 or control vehicle for another 30 min, then treated with amino acids for 30 min. Scale bar, 10 μm. **j** Co-IP assay showing the interaction of FLAG-mTOR and HA-TJP1 in primary hepatocytes cultured in a collagen sandwich configuration. Mouse primary hepatocytes were exposed to 250 nM Torin1 or control vehicle for 1 hr. **k** Immunoblots of co-IP assays showing the interaction of PER1 with WT TJP1 and mutants of TJP1 in HEK293T cells. For the amino acids marked in red, substitution of Serine (S) or Threonine (T) with either Alanine (A) or Aspartic acid (D) affects the interaction of TJP1 with PER1. **l** Amino acid sequence alignment of vertebrate TJP1 orthologs with the conserved mTOR phospho-sites marked in red. **m** Evaluation of phospho-TJP1 antibodies. *Tjp1* KO SD+ cultured primary hepatocytes were infected by adenovirus expressing GFP, WT TJP1, the 6A mutant or S1617A mutant of TJP1 for 2 days, and then incubated with amino acid-free RPMI1640 for 6 hrs before treatment with amino acids for 30 min. Data are shown as mean ± s.e.m. Comparison of different groups was carried out using one-way ANOVA (**e**) or two-way ANOVA (**b**). ***$P < 0.001$. Source data are provided as a Source Data file.

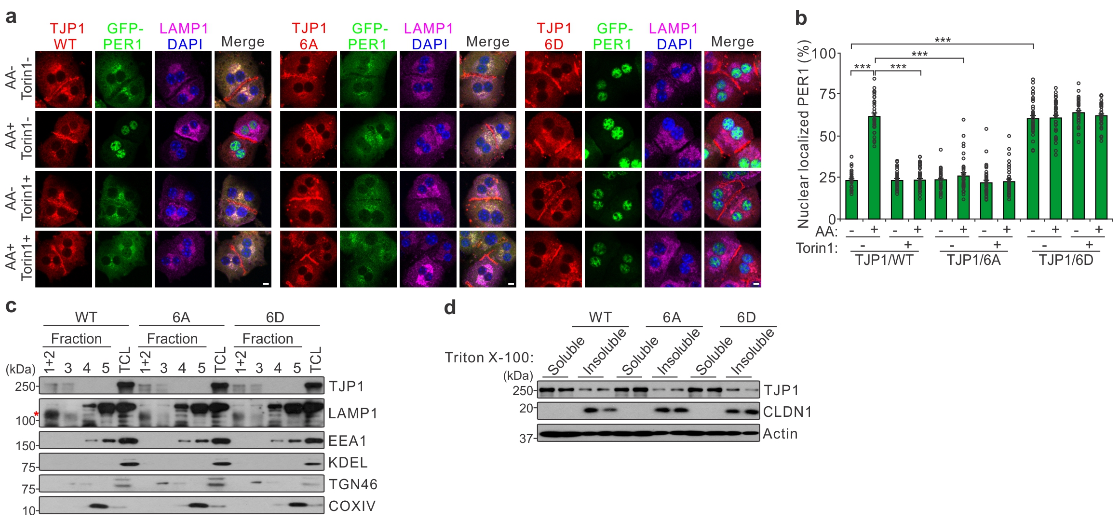

Supplementary Figure 4: Effect of TJP1 phosphorylation on PER1 nuclear translocation.

a-b, Cellular localization of GFP-PER1 (**a**) and statistical analysis of the results (**b**) showing the effect of TJP1 or its mutants on cellular localization of PER1 in mouse primary hepatocytes cultured in a collagen sandwich configuration. Mouse primary hepatocytes incubated with amino acid-free RPMI1640 for 6 hrs were exposed to 250 nM Torin1 or control vehicle for another 30 min, then treated with amino acids for 30 min. Scale bars, 10 μm. **c** Immunoblots showing the distribution of WT TJP1 and TJP1 mutants in lysosome fractions of mouse primary hepatocytes. The red asterisk indicates LAMP1. TCL, total cell lysate. **d** Immunoblots showing the relative distribution of WT TJP1 and TJP1 mutants in junction fraction (Triton X-100 insoluble) and cytosolic fraction (Triton X-100 soluble) of mouse primary hepatocytes. 6A is the T589A/T709A/S927A/S1360A/S1460A/S1617A mutant of human TJP1, while 6D is the T589D/T709D/S927D/S1360D/S1460D/S1617D mutant of human TJP1. Data are shown as mean ± s.e.m. Comparison of different groups was carried out using two-way ANOVA. ***P < 0.001. Source data are provided as a Source Data file.

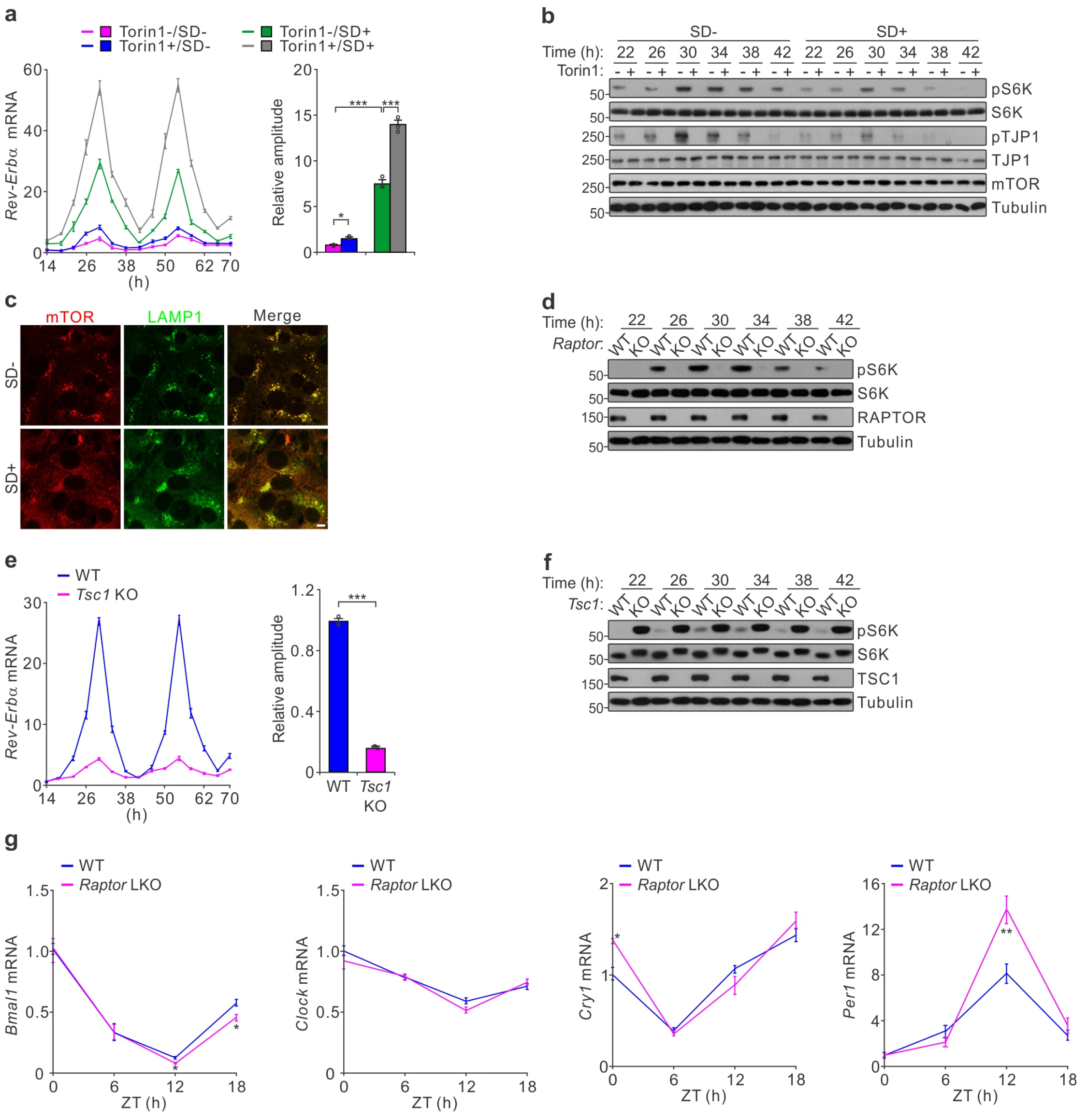

Supplementary Figure 5: mTOR decreases the circadian amplitude.

a qPCR results showing the relative mRNA level of *Rev-Erbα* (left panel) and rhythmic amplitude (right panel) in SD- or SD+ cultured primary hepatocytes in the presence or absence of torin1. Hepatocytes were exposed to dexamethasone (0.1 μM) with or without 250 nM Torin1 and then harvested at different time points. n = 6. **b** Immunoblots showing the amounts of pS6K and pTJP1 (S1614) in SD- or SD+ cultured primary hepatocytes in the presence or absence of torin1. **c** Immunostaining showing mTOR recruitment to the lysosome (LAMP1 staining) in SD- or SD+ cultured primary hepatocytes. Scale bar, 10 μm. **d** Immunoblots showing the amounts of pS6K in SD+ cultured WT or *Raptor* KO primary hepatocytes. **e** qPCR results showing the relative mRNA level of *Rev-Erbα* (left panel) and rhythmic amplitude (right panel) in SD+ cultured WT or *Tsc1* KO primary hepatocytes. Hepatocytes were exposed to dexamethasone (0.1 μM) and then harvested at different time points. n = 6. **f** Immunoblots showing the amounts of pS6K in SD+ cultured WT or *Tsc1* KO

primary hepatocytes. **g** qPCR results showing gene expression of core circadian oscillators in liver extracts from WT and *Tjp1* LKO mice. n = 4 ~ 7 mice. Data are shown as mean ± s.e.m. Comparison of different groups was carried out using two-way ANOVA. $^{*}P < 0.05$, $^{**}P < 0.01$, $^{***}P < 0.001$. Source data are provided as a Source Data file.

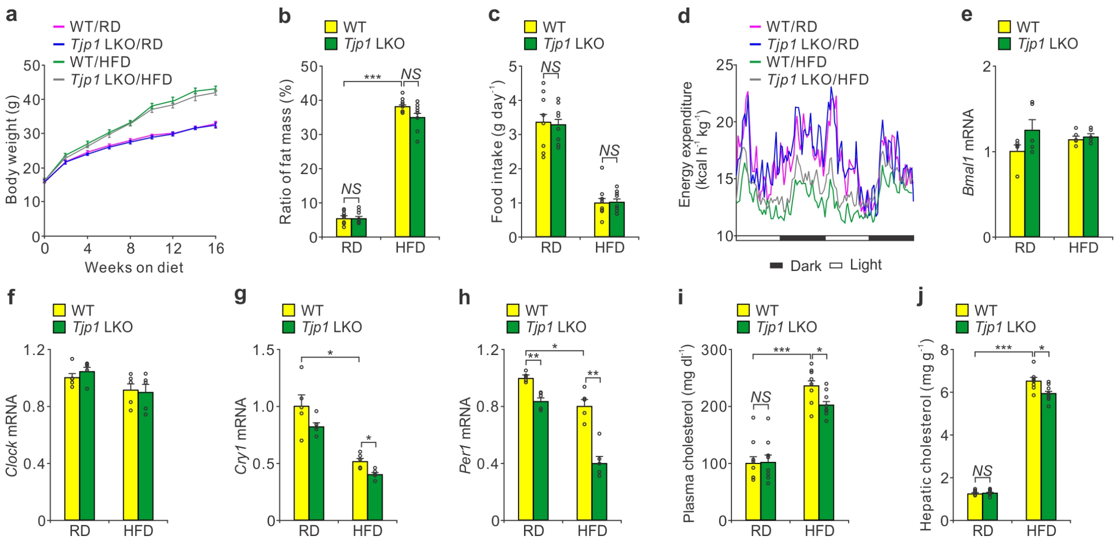

Supplementary Figure 6: Effect of *Tjp1* LKO on mouse metabolic parameters and circadian gene expression.

a-d Weight curves (**a**), ratio of fat mass (**b**), cumulative food intake (**c**) and energy expenditure (**d**) from WT and *Tjp1* LKO mice fed on a RD or HFD for 16 weeks. n = 8 ~ 10 mice. **e-h** Effect of *Tjp1* LKO on core circadian gene expression from mice fed on a RD or HFD for 16 weeks. n = 5 mice. **i-j,** Effect of *Tjp1* LKO on plasma cholesterol levels (**i**) and hepatic cholesterol contents (**j**) from mice fed on a RD or HFD for 16 weeks. n = 8 mice. Data are shown as mean ± s.e.m. Comparison of different groups was carried out using two-way ANOVA. **P* < 0.05, ***P* < 0.01, ****P* < 0.001. *NS*, no statistical significance. Source data are provided as a Source Data file.
